# An overview of the mental health system in Gaza: an assessment using the World Health Organization’s Assessment Instrument for Mental Health Systems (WHO-AIMS)

**DOI:** 10.1186/1752-4458-9-4

**Published:** 2015-01-16

**Authors:** Dyaa Saymah, Lynda Tait, Maria Michail

**Affiliations:** Primary Care Clinical Sciences, School of Health & Population Sciences, University of Birmingham, Edgbaston, Birmingham, B15 2TT UK; School of Health Sciences, University of Nottingham, Institute of Mental Health, Jubilee Campus, Triumph Road, Nottingham, NG7 2TU UK

**Keywords:** Mental health systems, Mental health, Global mental health, Policy, Legislation, WHO-AIMS, Gaza

## Abstract

**Background:**

Mental health system reform is urgently needed in Gaza to respond to increasing mental health consequences of conflict. Evidence from mental health systems research is needed to inform decision-making. We aimed to provide new knowledge on current mental health policy and legislation, and services and resource use, in Gaza to identify quality gaps and areas for urgent intervention.

**Methods:**

As part of a mixed methods study, we used the World Health Organization’s Assessment Instrument for Mental Health Systems Version 2·2 to collect data on mental health services and resources. Data collection was carried out in 2011, based on the year 2010.

**Results:**

Gaza’s mental health policy suggests some positive steps toward reform such as supporting deinstitutionalisation of mental health services. The decrease in the number of beds in the psychiatric hospital and the progressive transition of mental healthcare toward more community based care are indicative of deinstitutionalisation. However, mental health legislation in support of deinstitutionalisation in Gaza is lacking. The integration of mental health into primary healthcare and general hospitals has not been fully achieved. Mental health in Gaza is underfunded, human rights protection of service users is absent, and human resources, service user advocacy, and mental health training are limited.

**Conclusion:**

Priority needs to be given to human rights protection, mental health training, and investment in human and organisational resources. Legislation is needed to support policy and plan development. The ongoing political conflict and expected increase in need for mental health services demonstrates an urgent response is necessary.

## Introduction

Addressing the high global burden of mental disorders that are associated with substantial individual, social, and economic costs, especially in low- and middle-income countries (LMICs), and post-conflict areas, is an urgent priority [1, 2]. Epidemiological evidence that the mental health burden is higher in conflict areas of the world compared to regions with no conflict is compelling [3–7]. According to data on the effect of the prolonged Israel-Palestine conflict, 68.9% of adolescents exposed to ongoing conflict and violence in Gaza have developed post-traumatic stress disorder (PTSD), 40.0% moderate to severe levels of depression, and 94.9% severe anxiety [8]. PTSD is even higher in boys injured during Al-Aqsa intifada (2000–2007), 77% [9]. Although these prevalence estimates appear to be strikingly high, a systematic review of seventy-one eligible studies on the mental health of children and adolescents living in areas of armed conflict in the Middle East supports the high prevalence estimates, reporting PTSD to be between 23-70% in Palestine [10].

Mental illness contributes to a reduced quality of life and risk for early mortality [11]. In 2010, major depressive disorder rose by 37% of disability adjusted life years (DALYs) worldwide [12]. However, despite high levels of mental ill health and associated burden in LMICs, and post-conflict areas, and the fact that evidence-based, effective interventions can reduce this burden [13], treated prevalence rates in those areas are low, indicating a treatment gap and an urgent need for improvement in mental health care provision [14].

The landmark Lancet series on global mental health raised the profile of mental health systems in LMICs, with the aim to tackle the challenge of scaling up mental health services [1, 2]. Calls for global action included recommendations for research to assess mental health systems especially within LMICs to advance global mental health provision to meet the needs of populations [13, 15–17]. Yet limited systematic research has so far been conducted in LMIC and post-conflict areas.

Generating comprehensive baseline data on a country’s mental health system is essential to contribute to developing policy and plans to strengthen and scale up services [1]. This paper is based on the analysis of the WHO-AIMS survey [18] to provide a comprehensive overview of current mental health policy, legislation and services in Gaza to answer our research question: What are the current characteristics of the mental health system in Gaza in relation to policy development, service development and delivery, and availability of human resources?

## Methods

### Study area

The Gaza Strip is located in the Middle East, with an approximate geographical area of 365 square kilometres, bordered by 40 kilometres of the Mediterranean Sea, between Egypt and Israel. At the end of 2013, the population of Gaza was 1,730,737 people (879,158 males and 851,579 females), 43.3% of whom were below the age of 15 years [19]. Gaza is classified by the World Bank as a LMIC [20]. In 2013, the unemployment rate was 32.6% and 38.8% of the population in 2011 lived below the poverty line [19].

### Study design

This article presents results from a larger study using mixed methods to assess specific components of the mental health system in Gaza. The results reported here focus on an analysis of data collected using the World Health Organization’s Assessment Instrument for Mental Health Systems (WHO-AIMS) Version 2·2 [18].

### Instrument

The World Health Organization Assessment Instrument for Mental Health Systems (WHO-AIMS) questionnaire (Version 2·2) was used to conduct an assessment of the mental health system in Gaza based on the year 2010 [18]. The WHO-AIMS tool is an evidence-based tool [21], incorporating ten recommendations proposed by the World Health Report 2001 [22].

The WHO-AIMS questionnaire [18] has six domains: (1) policy and legislative framework, (2) mental health services, (3) mental health in primary healthcare, (4) human resources, (5) public education and links with other sectors, and (6) monitoring and research.

### Data sources

Purposive sampling was used to identify six informants, following the WHO-AIMS guidelines [18] that recommend selecting informants with access to all information that is needed to complete each of the six survey domains. Semi-structured interviews were conducted with four informants from the Ministry of Health, one informant from the Ministry of Education (mental health in schools), and one informant from the Islamic University of Gaza (mental health research).

### Data collection

Ethical approval was granted by the Helsinki Committee for Research Ethics in the MoH, Gaza, and the Ethical Review Committee, the University of Birmingham. Participants were provided with instructions to complete independently the WHO-AIMS questionnaire [18]. Data collection was carried out in 2011, and the first author completed the WHO-AIMS survey instrument [18] with data collected from the six key informants. To ensure data quality, returned completed surveys were checked with the six informants in face-to-face meetings to ensure data accuracy and consistency.

### Data analysis

All data were entered onto the WHO-AIMS standardised data spread sheet [18]. Descriptive statistical analyses were performed following the aggregation of numerical data. The final report on main findings conforms to the reporting guidelines of the WHO [22].

## Results

The current status of mental health policy, plans, services and resources are presented for each of the six domains of the WHO-AIMS instrument [18].

### Mental health policy and legislative framework

The Palestinian mental health policy was developed in 2004; last revised in 2010. The following components were included: (1) developing community mental health services, (2) downsizing mental hospitals, (3) developing a mental health component in primary healthcare, (4) human resources, (5) involvement of users and families, (6) advocacy and promotion, (7) human rights protection of users, (8) equity of access to mental health services, and (9) quality improvement. In addition, a list of essential medicines was present, including: (1) antipsychotics, (2) anxiolytics, (3) antidepressants, (4) mood stabilisers, and (5) antiepileptic drugs.

The mental health plan was drafted in 2010, consolidated and endorsed in 2011, and adopted by the Minister of Health in Gaza. This plan contains the same components as the mental health policy but also mentions reforming the mental hospital to provide more community-based services. There were well-defined goals and objectives and a timetable for implementing activities, but no budget was identified.

A draft Mental Health Act was developed in 2006 by one of the largest mental health non-governmental organisations (NGOs) in Gaza. The Legislative Council and the MoH have not approved it. Therefore, there was no mental health legislation in Gaza.

### Financing of mental health services

About 2% of the total healthcare budget was directed toward mental health by the MoH in Gaza. Of all expenditure spent on mental health, 56% was directed towards the mental hospital with 44% of the budget directed toward Community Mental Health Centres (CMHCs). At least 80% of essential psychotropic medicines are provided free of charge. However, when there are shortages in psychotherapeutic medications, the cost of private purchase of antipsychotic and antidepressant medication is 5% and 7% of the minimum daily wage in Gaza, respectively.

### Human rights policies

A national human rights review body does not exist in Gaza. This means that there are no inspection visits to mental health facilities and no resulting sanctions in cases of violation of service users’ rights. The mental hospital in Gaza did not receive any reviews/inspections of human rights for the protection of patients. Regarding training, none of the staff working in the mental hospital received at least one day of training, a meeting or other type of working session on human rights in the year this assessment took place.

### Mental health services

#### Organisation of mental health services

A National Mental Health Authority exists, which provides advice to the government on mental health policies and legislation. The Mental Health Authority is also involved in service planning and monitoring and quality assessment of mental health services. The mental health services are provided through outpatient services and one mental hospital. Mental health services are not organised into catchment/service areas.

There are many local and international NGOs in Gaza. The majority of NGOs provide a broad range of psychosocial, trauma-focused, programmes, while few provide specialised mental health services. The UN agencies and most international NGOs provide technical and financial support to the local government and local NGOs by supporting service development, staff training, and sometimes directing funds from international donors to local NGOs to support the implementation of projects. The WHO office in Gaza is an example of an international organisation that provides substantial financial and technical support to the Ministry of Health in support of mental health service development, especially the integration of mental health into PHC. Few international organisations provide direct service delivery to the population in Gaza, such as Save the Children and Médecins Sans Frontières. UNRWA is the largest service provider among international organisations. UNRWA provides psychosocial and mental health activities in 245 schools, 22 health centres, and 8 community rehabilitation centres.

### Mental health outpatient facilities

There were seven outpatient mental health facilities in Gaza, referred to as CMHCs. The first CMHC was established in 2004 and the last one in 2006. In 2010, those outpatient facilities treated 74·5 new service users per 100,000 population. Of all service users treated in CMHCs, 29% were female and 10% were children or adolescents. The service users treated in CMHCs were primarily diagnosed with neurotic disorders (18%), schizophrenia (14%), epilepsy (14%), mental retardation (13%), affective disorders (13%), organic disorders (7%), substance abuse disorders (4%), personality disorders (3%), and other mental disorders (14%). There was only one outpatient facility qualified to provide services to children and adolescents that represented 14% of outpatient services provided.

All CMHCs provided follow-up care in the community, while none of the facilities provided mobile mental health teams. Regarding available treatments in 2010, most patients (51-80%) in CMHCs received one or more psychosocial interventions. Cognitive behavioural therapy is provided by psychologists; recovery interventions by mental health nurses; psychosocial rehabilitation by mental health nurses and social workers; dialectical behavioural therapy by psychologists and mental health nurses; and psychological first aid by psychologists, social workers and nurses.

Moreover, all CMHCs had at least one psychotropic medicine from each therapeutic class (i.e. antipsychotics, antidepressants, mood stabilizers, anxiolytics, and antiepileptics) available in the facility or at a near-by pharmacy throughout the year.

### Mental hospital

There was one mental hospital available in Gaza, with 30 beds: 1·89 beds per 100,000 population. The number of beds decreased by 17% in the last five years. The hospital was integrated organizationally, with mental health outpatient facilities. There were no beds in the mental hospital reserved for children and adolescents only. The hospital treated 30·12 new users per 100,000 population. Among patients admitted to the hospital in 2010, 55% were females and no children or adolescents. The service users admitted to the mental hospital were primarily diagnosed with schizophrenia (69%) and mood disorders (14%); average length of stay was 8·68 days. The majority (80%) of service users spend less than one year in the mental hospital, 20% spend 5–10 years, and none spend more than ten years. In contrast to CMHCs, few service users (1-20%) in the mental hospital received one or more psychosocial interventions in the past year. The mental hospital had at least one psychotropic medicine of each therapeutic class available.

All the psychiatric beds in Gaza were located in the only mental hospital in Gaza City. The density of psychiatric beds in or around Gaza City is 2·94 times greater than the density of beds in the whole of the Gaza Strip. This distribution prevents equal access for the whole population in Gaza, especially for those living in rural areas.

### Mental health in primary healthcare

All facilities providing primary healthcare (PHC) in Gaza were physician-based. Of the 57 governmental PHC clinics in Gaza, almost all (81-100%) had assessment and treatment protocols for key mental health conditions; however, only a few (1-20%) made at least one referral per month to a mental health professional. PHC doctors are allowed to prescribe psychotropic medicines with restrictions. A few PHC clinics (1-20%) had at least one psychotropic medicine for most of the therapeutic categories (antidepressant, anxiolytic, and antiepileptic) available in the facility or at a near-by pharmacy all year long.

### Training of human resources in mental healthcare

Concerning undergraduate training for doctors and nurses in Gaza, 4% of the training for medical doctors was mental health related, in comparison to 7% for nurses, while non-doctor/non-nurse PHC workers received no mental health training. Only 30% of PHC doctors and 33% of nurses received at least two days of refresher training in mental health. It is notable that training of medical doctors and primary care nurses in mental healthcare is an ongoing process aimed at integrating mental health into PHC. Training was started in 2008 by the MoH, with support provided by the WHO.

### Human resources

The total number of healthcare workers in mental health facilities and private practice was 11·91 per 100,000 population as follows: 0·25 psychiatrists, 1·6 other medical doctors (not specialised in psychiatry), 4·8 nurses, 2·2 psychologists, 2·5 social workers, 0·5 occupational therapists, and 36·4 other health or mental health workers (including auxiliary staff, non- doctor/non-physician PHC workers, health assistants, medical assistants, professional and paraprofessional psychosocial counsellors). Table 1 displays the number of mental health professionals per professional group working within mental health facilities and private practice.

**Figure 1:**
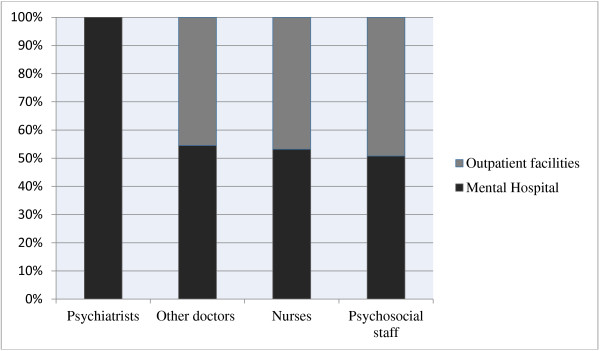
**Percentage of mental health staff according to place of work.**

Figure 1 shows the percentage distribution of governmental mental health workers between outpatient facilities and the mental hospital. There were 0·07 psychiatrists and 1·1 nurses per bed in the mental hospital. For other mental healthcare staff (e.g. psychologists, social workers, occupational therapists, other health or mental health workers), there were 1·03 staff per bed in the mental hospital.

**Table 1:** **Number of mental health professionals by discipline working within mental health facilities and private practice**

Mental health professional	Number of mental health professionals per 100,000
Psychiatrists	4
Medical Doctors	26
Psychologists	35
Social Workers	40
Nurses	76
Occupational Therapists	8
Other Mental Health Workers	577

The distribution of human resources between urban and rural areas was disproportionate. The density of psychiatrists and nurses in or around the largest city was 1·48 and 1.47 times greater than their density in all of Gaza, respectively.

### Training professionals in mental health

Table 2 displays the number of mental health professionals trained by academic institutions in Gaza per 100,000 population. It is notable that some psychiatrists (21-50%) emigrated to other countries within five years of completing their training. The number of doctors and nurses (not specialised in psychiatry) represents those who graduated from medicine and nursing schools in Gaza in 2010. The training they received in both schools was undergraduate training with no specialisation in mental health.

**Table 2:** **Number of trained mental health professionals per 100,000 population**

	Professionals graduate in mental health per 100,000
Psychiatrists	0
Medical Doctors	6·11
Nurses	11·7
Psychologists	1·57
Social Workers	0
Nurses + 1 year	1·57
Occupational Therapists	0

### Consumer and family associations

There was no official service user or carer associations in Gaza with legal or official representation in professional or legal activities. However, there were two advocacy groups for service users and carers organised by social workers in Gaza City and they met regularly in one of the CMHCs and the mental hospital in Gaza.

### Public education and links with other sectors

There was no coordinating body in Gaza to oversee public education and awareness campaigns on mental health. Government agencies, NGOs, and professional organisations all promoted public education and awareness campaigns in the last five years. These campaigns targeted the general population, women, trauma survivors, and other vulnerable groups. In addition, there were public education and awareness campaigns targeting professional groups, including teachers, healthcare providers, the complementary/alternative traditional sector, social services staff, leaders and politicians, and other professional groups linked to the health sector.

There were formal collaborations between the government department responsible for mental health and the departments/agencies responsible for PHC, child and adolescent health, substance abuse, child protection, education, welfare, and criminal justice. Concerning child and adolescent mental health, all primary and secondary schools had either a part-time or a full-time mental health professional, and almost all schools (51-80%) had school-based activities to promote mental health and prevent mental disorders. Regarding training, no police officers, judges, or lawyers had participated in educational activities on mental health in the last five years. Mental health facilities did not provide financial support to service users or link them to employment programmes outside mental health facilities.

### Monitoring and research

The mental health directorate received data from the only mental hospital in Gaza and all mental health outpatient facilities. However, no report was published on data transmitted to the mental health directorate. Research focused on epidemiological and non-epidemiological studies in community and clinical samples, services research and psychosocial, biology and genetics, and psychotherapeutic interventions.

## Discussion

This is the first study to report on current mental health policy, legislation, and services in Gaza, and provides a baseline for future progress and comparison with other countries. Our findings indicate some progress in mental health reform among many challenges, including a progressive transition of mental healthcare toward more community-based services, with a reduction in the number of hospital beds, and slowly integrating mental health into PHC. Nevertheless, the hospital consumed a large portion of the mental health budget and mental health staffing. This biased distribution of resources towards a mental hospital is common in most LMICs: funding from the mental health budget directed towards mental hospitals is 80% in Ghana [23–25], 55% in Uganda [26], and 67% in the whole world [27].

The integration of mental health into PHC began in Gaza in 2008, but by 2010 the provision of mental healthcare by PHC professionals was inadequate. Health planners and decision makers in Gaza need to continue the process of integrating mental health into PHC started by the MoH and the WHO. This integration could improve accessibility to mental health services by the population in Gaza, taking into consideration the shortage of specialised mental health professionals and the disproportionate distribution of mental health workers in rural areas.

Although the MoH started integrating mental health into PHC, mental health services need to be integrated into all health services. The study findings revealed the absence of community-based acute psychiatric units in general hospitals. The overdependence on the mental hospital in providing tertiary care could promote institutionalisation of mental health services and exhaust the financial and human resources allocated for mental health. The health authority in Gaza needs to promote the integration of mental health into secondary and tertiary health services and create more facilities for community-based rehabilitation in order to downsize the role of the mental hospital.

Other limitations of reform include a lack of mental health professionals, particularly psychiatrists who are key mental health service providers in a system dependent upon a biomedical approach to care, a lack of service user and carer representation in decision making or health planning activities, limited funding and human rights review bodies, and inadequate training of mental health staff.

The number of human resources in Gaza is comparable to many African countries [15]. Although the size of the mental health workforce in Gaza is higher than in low-income countries like Uganda [26], and Kenya [28], it is considerably lower than the number of other middle-income countries like Brazil [29], and Vietnam [30].

The lack of service user and carer participation in healthcare provision could lead to increased violation of human rights and discrimination of service users and carers. An empowering approach is needed if service users and carers are expected to contribute more effectively in providing suggestions for improvement or evaluating mental health services [31]. First, service providers need to improve communication with service users and carers, treat them as part of the decision-making process, and involve them in their care plans [32]. Second, the government needs to establish legal representation for service users and carers to ensure that they are represented in activities related to advocacy and policy and legislation development [33].

The absence of a Mental Health Act or any legislation mechanism for mental health practice in Gaza is in line with other developing countries without mental health legislation [17, 33], but contrasts with other Middle Eastern countries such as Saudi Arabia that has recently ratified their Mental Health Act [34]. There was an attempt to develop legislation in Gaza but this was not completed because of political factors. The lack of human rights monitoring and absence of legitimate service user and carer representation call for urgent action to be taken by the authority in Gaza to build upon the Mental Health Act developed in 2006 and to enhance mental health legislation to protect the human rights of service users and carers.

The study findings revealed under-spending on mental health services by the health authority in Gaza, consistent with lower than needed mental health spending in other LMICs [17]. Since 2004, international donors financially supported the transition of mental health services toward a community-based approach. However, this financial support is time limited and not sustainable [35]. Therefore, the health authority in Gaza needs to increase their spending on mental health to sustain and expand the development of a community-based approach to mental health services.

Mental health reform in conflict and post-conflict countries is affected by the consequence of conflict on prioritising the health agenda. It is rare to find mental health reform at the top of the health planners’ agenda in areas affected by emergency situations [36]. Contributory factors to this low priority are that unstable security situations discourage donors and policy makers from supporting the long-term development of mental health systems [37], and the tendency of policy makers to address more existential concerns that do not include mental health reform, which was the case in Israel [38]. Consequently, although the mental health burden in post-conflict areas is higher than in more stable countries [6, 7, 39], mental health services in most low-resourced and conflict-affected countries are still under-resourced and insufficient to respond to such high needs [16, 17].

One of the main barriers toward developing mental health resources in post-conflict areas, and LMICs, is the low governmental spending on developing mental health services, which are biased toward institutionalised medical services. Although the mental health policy, plan and legislation were well-developed in Ghana, mental health services were also underfunded: only 1.4% of the health expenditure was spent on mental health [24]. The assessment of the mental health system in Ghana revealed a broad provision of mental health services in outpatient services, mental hospitals, community-based psychiatric units, residential facilities, and day treatment centres. However, the number of mental health workers was extremely unbalanced toward medical staff. For example, there were 19 psychologists compared to 1,068 mental health nurses [24]. Similarly, Uganda has taken substantial steps toward decentralisation of mental health services. However, the governmental spending on mental health services does not exceed 1% of the governmental health budget and 55% of this fund was spent on mental hospitals [26].

Although resources for reforming mental health services in post-conflict areas are insufficient, there are positive examples of improving accessibility to mental health services: Darfur has successfully integrated the management of five common mental disorders into PHC [40] and, although spending on mental health services in Uganda was insufficient, mental health services were broadly integrated into PHC and general hospitals [26]. The experience of integrating mental health services in PHC in Darfur and Uganda demonstrates the potential for the decentralisation of mental health services in low-income, post-conflict settings.

Substantial progress has been achieved toward integrating mental health into PHC in Gaza. The MoH, supported by the WHO, is implementing a district level, stepped-care programme, aiming to integrate mental health into all 54 governmental PHC centres. At least 50% of this target has been achieved to date. One service user and carer organisation has been established: it is poorly funded, and more focused on advocacy and awareness raising, but its role in policy and service development is still uninfluential. The number of beds in the mental hospital was decreased from 30 to 24. One day care centre has been created inside the mental hospital in 2014; its focus is on occupational therapy and recovery, for people with severe mental illnesses.

### Study limitation

The WHO-AIMS questionnaire [18] should be completed by key informants appointed from relevant organisations. Although participants in our study were relevant to completing the questionnaires, many questions required professional judgment on the current situation of service development. A potential limitation of this study, therefore, is that a single person judgment can potentially introduce bias as this judgment could reflect personal views and attitudes. Our solution was to check and confirm the accuracy and source of information with all six participants in face-to-face meetings.

## Conclusion

The mental health system in Gaza has achieved substantial progress toward the de-institutionalisation of mental healthcare; however, many challenges remain. The ongoing political conflict in Gaza and associated increase in the need for mental health services should put more pressure on authorities in Gaza to invest more resources in mental health.

Establishing community mental health centres and downsizing the mental hospital should improve accessibility to mental health services in primary care and general hospitals. To achieve this, authorities in Gaza need to increase expenditure on mental health, and increase the number of skilled mental health professionals. Mental health policy and service development in Gaza should consider service user and carer human rights. This can only be achieved by developing mental health legislation to enhance mental health policy implementation, and by promoting service user and carer participation in all levels of policy and service development.

This study adds to the limited research on mental health reform in LMICs and post-conflict areas and provides important information on progress and gaps to inform policy makers and health planners on the distribution of scarce resources and priority areas for urgent intervention. Furthermore, the findings of the current study add to the knowledge base in developing mental health services in LMICs, and especially countries affected by conflict, by highlighting common gaps and the need for better use of available resources.

## Authors’ information

DS is a Researcher at the World Health Organization and is completing his Doctoral studies at the University of Birmingham. He has a Masters degree in Psychology from The Islamic University in Gaza.

LT is a Chartered Scientist and Chartered Psychologist, and is currently a Researcher at Nottingham University and an Honorary Research Fellow at the University of Birmingham. She holds a PhD in Psychology from the same university.

MM is a Senior Research Fellow at the University of Nottingham and she holds a PhD in Psychology from the University of Birmingham.
